# Identification and validation of a novel locus, *Qpm-3BL*, for adult plant resistance to powdery mildew in wheat using multilocus GWAS

**DOI:** 10.1186/s12870-021-03093-4

**Published:** 2021-07-30

**Authors:** Xijun Du, Weigang Xu, Chaojun Peng, Chunxin Li, Yu Zhang, Lin Hu

**Affiliations:** 1 College of Agronomy, Northwest A&F University, Yangling, Shanxi 712100 Xianyang, China; 2 Institute of Crop Molecular Breeding/National Engineering Laboratory of Wheat/Key Laboratory of Wheat Biology and Genetic Breeding in Central Huanghuai Area/Ministry of Agriculture/Henan Key Laboratory of Wheat Germplasm Resources Innovation and Improvement, Henan Academy of Agricultural Sciences, 450002 Zhengzhou, China

**Keywords:** Wheat, Powdery mildew, Adult plant resistance, 660K microarray, Multilocus genome-wide association study, *PmBMYD*, Marker-assisted breeding

## Abstract

**Background:**

Powdery mildew (PM), one of the major diseases in wheat, severely damages yield and quality, and the most economical and effective way to address this issue is to breed disease-resistant cultivars. Accordingly, 371 landraces and 266 released cultivars in Henan Province were genotyped by a 660 K microarray and phenotyped for adult plant resistance (APR) to PM from 2017 to 2020, and these datasets were used to conduct multilocus genome-wide association studies (GWASs).

**Results:**

Thirty-six varieties showed stable APR in all the environments, and eleven quantitative trait nucleotides (QTNs) were found by multiple methods across multiple environments and best linear unbiased prediction (BLUP) values to be significantly associated with APR. Among these stable QTNs, four were previously reported, three were newly discovered in this study, and the others need to be further investigated. The major and newly discovered QTN, *Qpm-3BL*, was located at chr03BL_AX-109,052,670, while another newly discovered QTN, *Qpm-1BL*, was located between chr01BL_AX-108,771,002 and chr01BL_AX-110,117,322. Five and eight landraces were identified to be resistant based on *Qpm-1BL* (haplotype TC) and *Qpm-3BL* (allele T), respectively. To validate *Qpm-3BL*, a new kompetitive allele-specific PCR (KASP) marker was developed to scan 155 F_2_ individuals, and the average resistance score supported the value of *Qpm-3BL* in marker-assisted breeding. Near *Qpm-3BL*, *PmBMYD* was identified by KEGG, gene expression and comparative genomics analyses to be a candidate. Its resistance mechanism may involve gene tandem repeats.

**Conclusions:**

This study reveals a previously unknown gene for PM resistance that is available for marker-assisted breeding.

**Supplementary Information:**

The online version contains supplementary material available at 10.1186/s12870-021-03093-4.

## Background

As one of the most important staple crops, wheat is grown on approximately 17 % of arable land and feeds nearly 40 % of the global population [1]. Powdery mildew (PM), a worldwide leaf disease caused by *Blumeria graminis* f. sp. *tritici* (Bgt), occurs mainly in cold and humid areas, such as China, Europe, and the Southern Cone of South America [2]. PM can reduce wheat yield by 5–62 % and severely affect flour quality [3, 4]. With the popularization of dwarf and semidwarf wheat cultivars and the improvement in water and fertilizer conditions, more favorable conditions have been created for the occurrence and spread of PM in wheat, and the incidence area has gradually expanded. Although the use of pesticides is currently the main method to prevent diseases, it also accelerates the variation in physiological races of pathogens and makes the problem more complicated. The most economical, green and effective way to address this issue is to breed disease-resistant cultivars.

According to wheat resistance to PM, disease resistance genes can be divided into qualitative and quantitative resistances or seedling and adult resistances. Qualitative resistance is a high level of resistance to one or more physiological races without resistance to other physiological races during the whole growth period, which is consistent with the “gene-to-gene” hypothesis proposed by Flor [5, 6]. Adult resistance, known as partial resistance, is stable and persistent resistance to all physiological races [7, 8]. Among the 68 known wheat PM resistance genes (*Pm1*-*Pm68*) [9], most are qualitative resistance genes, except for *Pm38* [10], *Pm39* [11] and *Pm46* [12]. However, most of them lost their resistance function rapidly after the variation in their physiological races [12, 13]. Compared with the application of cultivars containing only a qualitative resistance gene, breeding new wheat varieties with quantitative PM resistance genes/quantitative trait loci (QTLs) has been shown to be more effective in controlling PM isolates [14].

Before the 21st century, the discovery of PM resistance genes in common wheat was focused mainly on bred cultivars and their fundamental parents. Due to the similar genetic basis of these cultivars, it is becoming increasingly difficult to discover new PM resistance genes. However, landraces have relatively extensive genetic diversity, and they carry abundant PM resistance genes [15]. To date, at least 18 PM resistance genes have been identified in Chinese landraces. Among these resistance genes, nine are located on 7BL, namely, *Pm5d* [16], *Pm5e* [17], *MLxbd* [18], *PmH* [19], *MLmz* [20], *PmHYM* [21], *PmBYYT* [22], *PmSGD* [23], and *PmTm4* [24]; three are on 1DS, namely, *Pm24a* [25], *Pm24b* [26], and *PmHLT* [27]; and *PmX* [28], *Pm61* [29], *pmQ* [30], *Pm47* [31], *Pm2C* [32] and *Pm45* [33] are located on 2AL, 4AL, 2BL, 7BS, 5DS and 6DS, respectively. China is the largest contributor to wheat production in the world. Henan is the main wheat-producing province in China; its perennial planting area accounts for approximately 23 % of the total wheat planting area in China, and its total output accounts for approximately 27 % of the total wheat production in China. At present, almost all wheat PM resistance gene mining in Henan Province has been conducted in biparental segregation populations, but disease resistance genes in wheat germplasms have not been fully explored.

To address the above issues, in this study, 637 core wheat germplasms in Henan Province were genotyped by a 660 K microarray and phenotyped by monitoring adult plant resistance (APR) to PM from 2017 to 2020. These accessions included 371 ancient landraces and 266 released cultivars, which have been widely popularized cultivars, fundamental parents and current budding cultivars for the past 70 years. These datasets were used to perform a multilocus genome-wide association study (GWAS) via mrMLM software [34], and the purpose was to identify elite wheat accessions with APR to PM and mine their resistance genes for wheat resistance breeding.

## Results

### Screening for PM-resistant wheat accessions

A set of 637 wheat accessions was collected from Henan Province in China. Their APR scores were measured at the adult plant stage in the field from 2017 to 2020. These scores ranged from 0 to 4, and the percentage of resistant accessions (PM score ≤ 2) varied from 9.26 to 15.86 % in all the field tests. The broad-sense heritability of resistance to PM was 0.76, indicating the existence of large genetic variation for this trait. This screening led to the identification of 36 (5.65 %) consistently resistant accessions, including 21 landraces and 15 released cultivars (Additional file 1: Table S1).The high correlations (r^2^ = 0.6258–0.9044; P-value < 0.01) of the PM scores across various field environments indicated the high quality of the datasets (Additional file 1: Table S2).

### Genotypic data, LD and population structure analysis

A total of 637 wheat varieties were genotyped by 660 K single-nucleotide polymorphism (SNP) genotyping arrays. After filtering, a total of 314,548 high-quality SNP markers were retained and used in this study. These SNP markers were located on all 21 wheat chromosomes. The number of SNPs varied across chromosomes, with a minimum of 2270 SNPs on chromosome 4D (chr-4D) and a maximum of 37,011 SNPs on chr-3B. In addition, the numbers of SNPs varied across subgenomes, with a maximum of 144,945 SNPs on subgenome B, followed by subgenome A (134,300), and a minimum of 35,303 SNPs on subgenome D (Table 1). These results are consistent with results reported in the literature [35, 36]. The abovementioned high-quality SNP markers were used to calculate linkage disequilibrium (LD) decay. Based on the critical r^2^ value of 0.2, the LD decay distance was approximately 16.89 Mb (Additional file 2: Figure S1).

**Table 1:** Information on the SNPs on the 21 wheat chromosomes in 637 accessions

Chr	No. of SNPs	Chr size (Mb)	SNP coverage	Chr	No. of SNPs	Chr size (Mb)	SNP coverage	Chr	No. of SNPs	Chr size (Mb)	SNP coverage ^a^
1 A	23,719	594.10	39.92	1B	14,912	830.83	17.95	1D	6194	566.08	10.94
2 A	22,941	689.85	33.26	2B	22,745	615.55	36.95	2D	6027	618.08	9.75
3 A	15,925	495.45	32.14	3B	37,011	744.59	49.71	3D	4437	720.99	6.15
4 A	18,346	780.80	23.50	4B	9978	673.62	14.81	4D	2270	473.59	4.79
5 A	17,917	801.26	22.36	5B	26,236	509.86	51.46	5D	4800	736.71	6.52
6 A	12,682	651.85	19.46	6B	21,304	709.77	30.02	6D	4676	750.62	6.23
7 A	22,770	750.84	30.33	7B	12,759	713.15	17.89	7D	6899	638.69	10.80
Total	134,300	4764.15			144,945	4797.37			35,303	4504.76	

^a^*SNP coverage* the number of SNPs per Mb in chromosomes

**Fig. 1:**
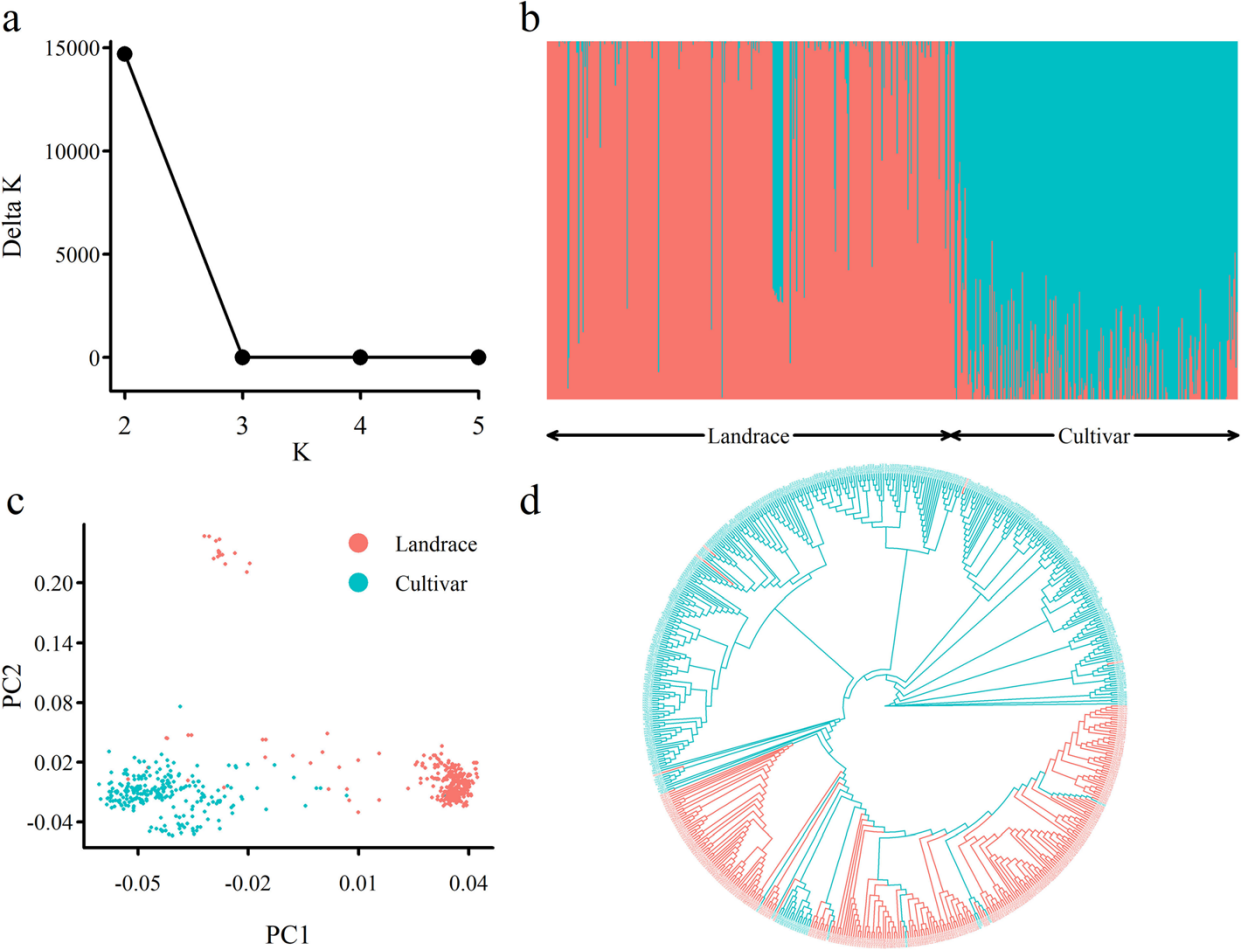
Population structures of 637 wheat accessions. (**a**) Determination of the number of subpopulations via the ad hoc statistic $\Delta D$. (**b**) Structure analysis. The 1st subpopulation mainly consisted of landraces, while the 2nd subpopulation mainly consisted of released cultivars. (**c**) PCA. (**d**) Neighbor-joining tree analysis. In the phylogenetic tree, most landraces were clustered into one cluster, while most cultivars were clustered into another cluster

Analysis of population structure for the 637 wheat accessions based on the 314,548 SNPs showed that the maximum peak value of $\Delta D$ appeared at *K* = 2 when the number of subpopulations increased from 2 to 5 (Fig. 1a, b). When *K* = 2, ancient landraces and released cultivars were separated. In the first subpopulation (354 accessions), 345 were landraces, and 9 were released cultivars. In the second subpopulation (283 accessions), 256 were released cultivars, and 27 were landraces (Additional file 3). To further confirm the abovementioned population structure, phylogenetic tree construction and principal component analysis (PCA) were carried out. Similar results were observed by these analyses (Fig. 1c, d).

### Multilocus GWAS for APR to PM in 637 wheat accessions

**Fig. 2:**
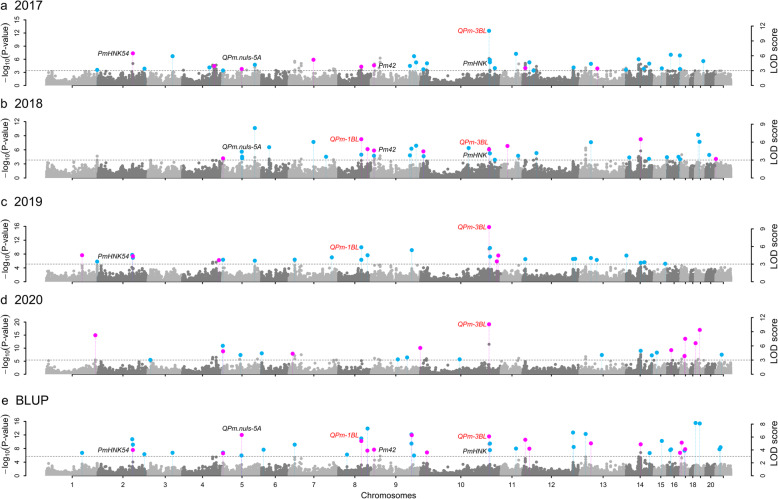
Manhattan plots for wheat APR to PM in 2017 (**a**), 2018 (**b**), 2019 (**c**), and 2020 (**d**) and their BLUP values (**e**). The horizontal line indicates a critical LOD score of 3.0. Chromosomes 1–7: chromosomes 1-7 A; chromosomes 8–14: chromosomes 1B-7B; chromosomes 15–21: chromosomes 1D-7D. Genes indicated in black text have been reported in previous studies and in the current study, and genes indicated in red text were newly identified in this study. Significant sites in blue and purple represent SNP markers that be detected by one or more methods, respectively

Using six multilocus GWAS methods in mrMLM software, the PM scores of 637 wheat accessions from 2017 to 2020 along with their best linear unbiased prediction (BLUP) values were used for association with the 314,548 high-quality SNPs. A total of 100 SNPs on all 21 chromosomes except for chr-4D and chr-6D were found to be significantly associated with the PM scores in all four field environments (Additional file 2: Figure S2, Additional file 1: Table S3). Among these quantitative trait nucleotides (QTNs), eleven were identified by various methods in at least two environments and the BLUP values (Fig. 2; Table 2, Additional file 4), and thirteen were on chr-3B. Haplotype analysis for all 13 QTNs on chr-3B showed that chr03BL_AX-109,052,670 and chr03BL_AX-111,134,486 were not in the same LD block (Additional file 2: Figure S3a), indicating the existence of two loci (a new locus and *PmHNK*). Among these stable QTNs, chr02AL_AX-110,404,341, chr05AL_AX-111,769,070, chr02BS_AX-110,111,855 and chr03BL_AX-111,134,486 were reported in previous studies to be closely linked to *PmHNK54* [37], *Qpm.nuls-5 A* [11], *Pm42* [38] and *PmHNK* [39], respectively. Here, we focused on the previously unknown QTNs. First, one newly discovered QTN (chr03BL_AX-109,052,670) on chr-3BL was detected 12 times by five methods in all four environments and by the BLUP values, which we named *Qpm-3BL*, and the proportion of total phenotypic variance explained by this QTN was 0.00-12.98 %. Then, we focused on two new SNPs, namely, chr01BL_AX-110,117,322 and chr01BL_AX-108,771,002, which were identified two (r^2^ = 1.27–3.35 %) and six (r^2^ = 0.00-2.13 %) times, respectively (Table 2, Additional file 4). Owing to the only 100 kb distance between the two SNPs, haplotype analysis was also conducted. The two SNPs were located on a strong LD block (Additional file 2: Figure S3b); thus, the two QTNs should constitute one locus, which we named *Qpm-1BL*.

**Table 2:** Eleven stable QTNs identified by multilocus GWAS methods in four environments and their BLUP values

SNP	Chr	Position (bp)	LOD score	r^2^ (%)	Repeatability across environments/methods			R/S allele	Comparative genomics analysis		
					**Environment**	**Method**^**a**^	**Times**		**Linked genes/QTLs**^**b**^	**Annotation**	**Distance (kb)**
AX-110,404,341	2 A	694,749,410	3.54–8.02	2.25–4.66	2017, 2019	3, 5, 6	6	T/G	*PmHNK54*	-	-
AX-108,822,998	5 A	5,704,663	3.21–6.43	1.13–3.68	2018–2020	3 ~ 5	5	T/C	*TraesCS5A01G007900.1*	Lipoxygenase	745
AX-111,769,070	5 A	502,896,109	3.11–4.40	1.58–2.19	2017, 2018	2, 3, 6	3	C/G	*QPm.nuls-5 A*	-	-
AX-108,771,002	1B	582,441,405	3.73–3.90	0-2.13	2018, 2019	1, 2	2	C/T	*TraesCS1B01G604700LC.1*	NBS-LRR like	145
AX-110,117,322	1B	582,542,111	3.52–8.37	1.27–3.35	2017–2019	3 ~ 6	6	C/A			
AX-110,111,855	2B	26,097,938	3.37–5.82	1.07–3.04	2017, 2018	2, 3, 5, 6	6	G/A	*Pm42*	-	-
AX-109,052,670	3B	730,604,593	3.22–20.18	0-12.98	2017–2020	1, 2, 4 ~ 6	12	T/C	*TraesCS3B01G483700.1*	NBS-LRR	292
AX-111,134,486	3B	738,687,251	4.12–4.70	1.64–1.99	2017, 2018	2, 3	2	C/T	*PmHNK*	-	-
AX-110,494,139	5B	27,356,806	3.28–3.84	0.39-2.00	2017, 2019	2, 4	3	G/T	*TraesCS5B01G027700.1*	NBS-LRR like	477
									*TraesCS5B01G029100.1*	Serine/threonine-protein phosphatase	473
AX-109,883,927	6B	159,311,957	4.04-6.00	1.42–2.81	2017–2019	4, 6	3	C/A	*TraesCS6B01G156800.2*	Leucine-rich repeat receptor-like protein kinase family protein	66
									*TraesCS6B01G157000.1*	Leucine-rich repeat receptor-like protein kinase family protein	90
									*TraesCS6B01G157700.1*	Pathogenesis-related thaumatin family protein	640
									*TraesCS6B01G157800.1*	Leucine-rich repeat receptor-like protein kinase family protein	676
AX-108,787,526	7B	405,972,463	3.24–9.91	1.76–4.46	2018–2020	1, 4 ~ 6	6	G/A	*TraesCS7B01G395600LC.1*	NAC domain protein	393
									*TraesCS7B01G395900LC.1*	Catalase-peroxidase	86

^a^ The six methods mrMLM, FASTmrMLM, FASTmrEMMA, pLARmEB, pKWmEB, and ISIS EM-BLASSO are marked as 1 to 6, respectively

^b^ Potential candidate genes were selected based on gene function annotation

**Fig. 3:**
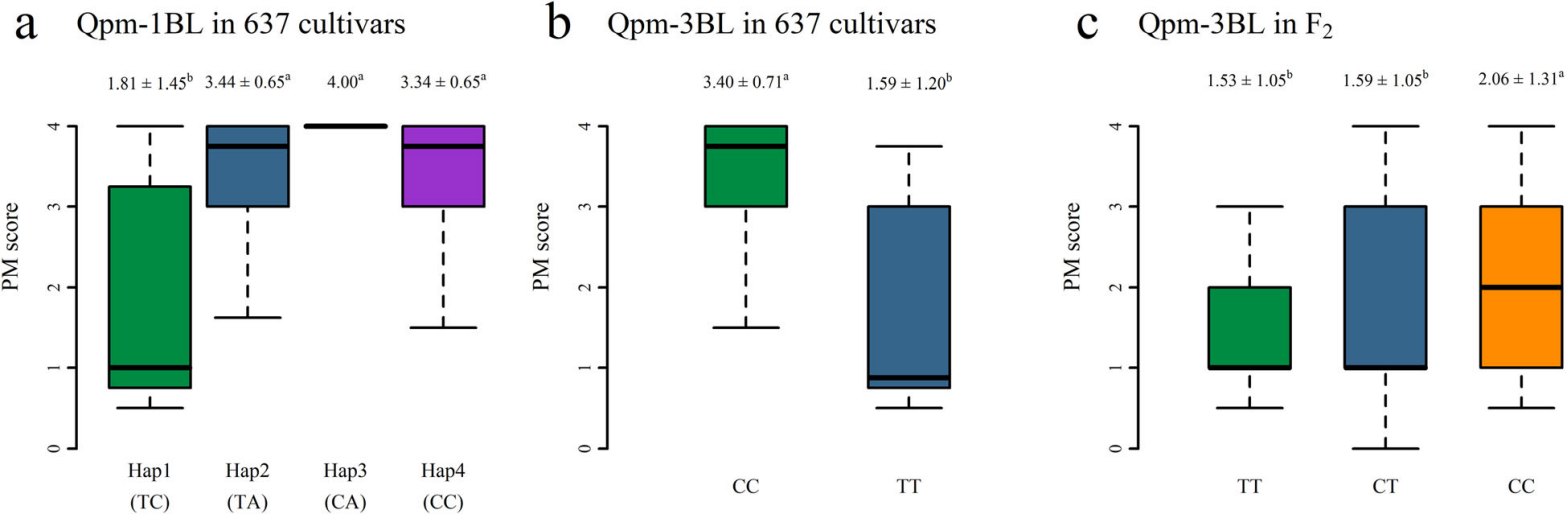
PM scores of *Qpm-1BL* (**a**) and *Qpm-3BL* (**b**) in 637 wheat accessions and of *Qpm-3BL* (**c**) in 155 F_2_ individuals

To further understand *Qpm-1BL* and *Qpm-3BL* in this study, we calculated the distribution frequencies and resistance levels of different haplotypes for these two loci in the 637 accessions. For *Qpm-1BL*, all 637 accessions were grouped into four haplotypes (the resistance haplotype TC and sensitivity haplotypes TA, CA and CC), and the differences in the average PM score across the four haplotypes were very significant (P-value < 0.01). Eight accessions had the favorable resistance haplotype TC, the average PM score was 1.81 ± 1.45 in all four environments, and five landraces were resistant; 242 accessions had the susceptibility haplotype TA, and the average PM score was 3.44 ± 0.65; one accession had the susceptibility haplotype CA, and its PM score was 4.0; and 386 accessions had the susceptibility haplotype CC, and the average PM score was 3.34 ± 0.65 (Fig. 3a). For *Qpm-3BL*, all 637 accessions were grouped into two haplotypes (resistance allele T and sensitivity allele C), and the difference in the average PM score between the two haplotypes was very significant (P-value < 0.01). Twelve accessions had the favorable resistance allele T, the average PM score was 1.59 ± 1.20 in all four environments, and eight landraces were resistant, whereas 625 accessions had the sensitivility allele C, and the average PM score was 3.40 ± 0.71 in all four environments (Fig. 3b).

### Validation of ***Qpm-3BL*** in an F_2_ population

To further validate *Qpm-3BL*, the SNP chr03BL_AX-109,052,670 was converted to a kompetitive allele-specific PCR (KASP) marker, and the resistant accession Baimayidan (haplotype TT) was crossed with the susceptible accession Pumai 9 (haplotype CC) to obtain 155 F_2_ plants; these F_2_ individuals were scanned with the new KASP marker. As a result, the number of plants with the marker genotypes TT, TC and CC in F_2_ were 46 (35R:11 S), 69 (51R:18 S) and 40 (24R:16 S) (Additional file 5), respectively. The segregation ratio fit 1:2:1 (χ^2^ = 2.329, P-value = 0.3121), and the resistance penetrance decreased from 0.76 for haplotype TT to 0.60 for haplotype CC. The average resistance scores were 1.53 ± 1.05 for TT, 1.59 ± 1.05 for TC, and 2.06 ± 1.31 for CC (P-value < 0.05) (Fig. 3c). This finding indicates that the KASP marker is available for marker-assisted breeding for resistance to PM in wheat.

### Identification of candidate genes linked with ***Qpm-3BL***

To identify the candidate genes for *Qpm-3BL*, a total of 68 genes were found to be within the 750 kb sequence on either side of the SNP chr03BL_AX-109,052,670 by using pairwise LD correlations (r^2^ ≥ 0.5). These genes were used to conduct KEGG analysis on the website KOBAS 3.0 (http://kobas.cbi.pku.edu.cn/kobas3/?T=1). As a result, 8 significant pathways were found to be enriched (Table 3). Among these pathways, the plant-pathogen interaction pathway with two genes, namely, *TraesCS3B01G483600.1* and *TraesCS3B01G483700.1*, was annotated by IWGSC RefSeqv1.0 (http://202.194.139.32/) as containing nucleotide-binding site-leucine-rich repeat (NBS-LRR) plant disease resistance genes. To further examine the candidates, we first downloaded expression datasets for the 68 candidate genes for 7-day seedlings of accession N9134 (resistant) from expVIP (http://www.wheat-expression.com/) [40]. Genes with expression levels less than 0.06 transcripts per million (tpm) were regarded as nonexpressing (0 tpm). No expression at 0, 24, 48 and 72 h was indicated by a value of 0 tpm, while the relative expression levels at 24, 48 and 72 h for the other genes were calculated from their corresponding real expression levels at 0 h. Except for *TraesCS3B01G708400LC.1*, which was expressed at only 0.12 tpm at 72 h and was not expressed at other times, the expression of all the candidate genes is shown in a heat map produced by TBTools v1.082 software (Additional file 2: Figure S4). The results showed that the expression levels of *TraesCS3B01G483600.1* and *TraesCS3B01G483700.1* at 48 h after inoculation with the wheat PM pathogen E09 were approximately two times higher than those before inoculation. Using NCBI BLAST (https://blast.ncbi.nlm.nih.gov/Blast.cgi), we then identified genes homologous to the above two genes. Using DNAMAN v9.0 software, we found one homologous gene, *RGA S-L8* (GenBank:AJ507098.1), which was previously reported to be involved in PM resistance in barley [41, 42], and the protein identities were 43.48 and 94.17 % (Additional file 2: Figure S5), respectively. In conclusion, *TraesCS3B01G483700.1* was selected as the preferred candidate gene for *Qpm-3BL* and designated *PmBMYD*.

**Table 3:** KEGG pathway analysis for 68 genes near Qpm-3BL

KEGG pathway	ID	Input number	Background number	P-value	Corrected P-value
Cyanoamino acid metabolism	osa00460	8	54	2.34E-13	1.85E-11
Phenylpropanoid biosynthesis	osa00940	10	233	2.15E-11	8.49E-10
Starch and sucrose metabolism	osa00500	8	161	8.31E-10	2.19E-08
Metabolic pathways	osa01100	19	2290	1.27E-08	2.51E-07
Biosynthesis of secondary metabolites	osa01110	14	1177	2.16E-08	2.84E-07
Tropane, piperidine and pyridine alkaloid biosynthesis	osa00960	4	28	3.47E-07	3.43E-06
Oxidative phosphorylation	osa00190	5	147	8.79E-06	2.57E-05
**Plant-pathogen interaction**	osa04626	2	168	0.038779	0.04031

### Gene tree and motif analysis of PmBMYD in different plants

**Fig. 4:**
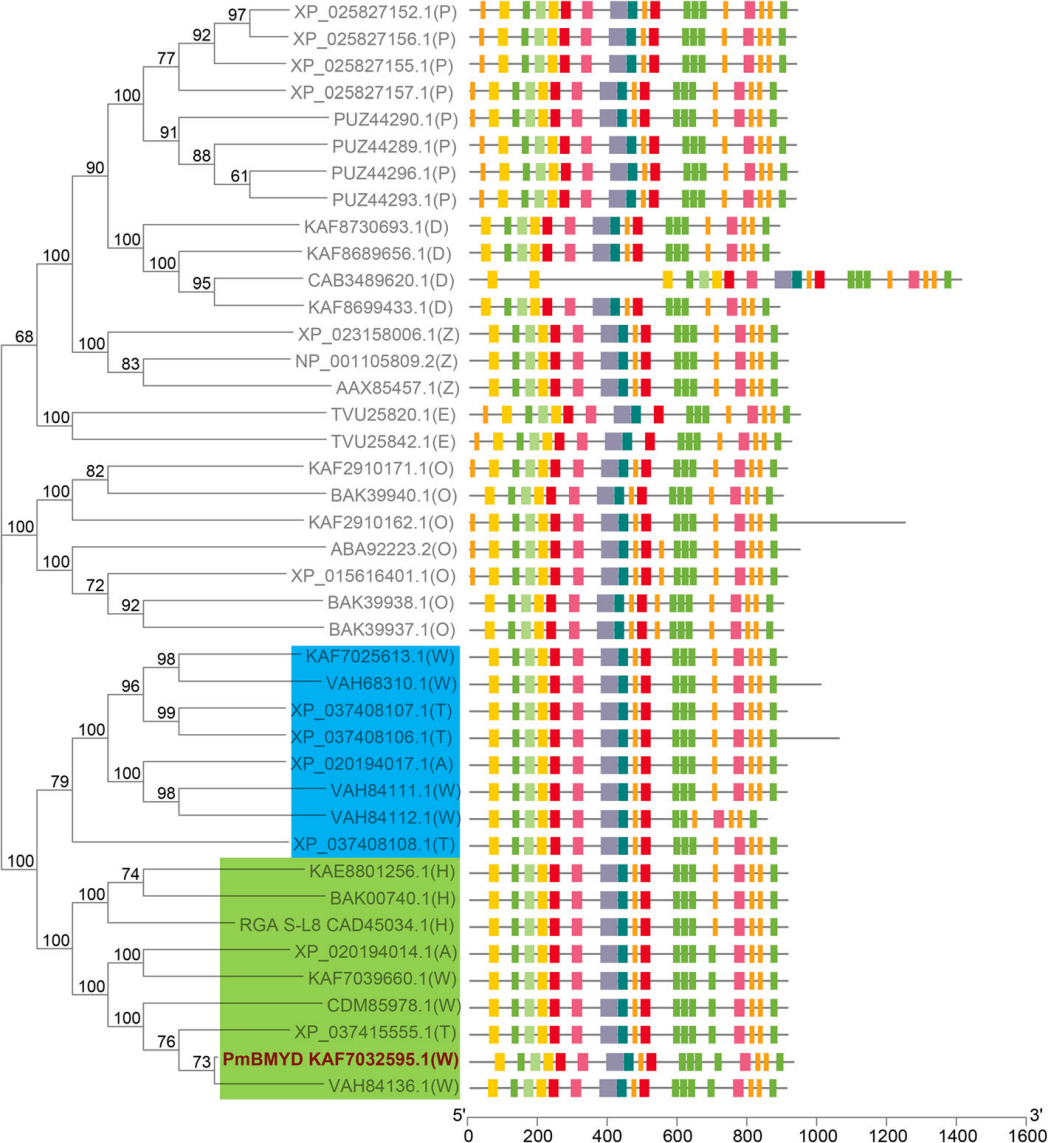
Gene tree and motif analysis of *PmBMYD* in different plants. W: common wheat; T: *Triticum dicoccoides*; A: *Aegilops tauschii*; H: *Hordeum vulgare*; O: *Oryza*; Z: *Zea mays*; P: *Panicum hallii*; E: *Eragrostis curvula*; D: *Dichanthelium exilis*

The protein sequences encoded by *PmBMYD* were searched in the NCBI database by BLASTP, and the top 100 homologous proteins were obtained from common wheat (16), *Hordeum vulgare* (6), *Oryza* (22), *Panicum hallii* (8), *Zea mays* (8), sorghum (2), *Aegilops tauschii* (4), *Dichanthelium exilis* (6), *Foxtail millet* (4), *Panicum miliaceum* (3), *Saccharum hybridcultivar* (1), *Setaria viridis* (4), *Brachypodium distachyon* (1), *Triticum dicoccoides* (8), *Triticum urartu* (2) and *Eragrostis curvula* (3). The protein sequence of *PmBMYD* was the same as that of the hypothetical unknown-function protein *CFC21_043753* (accession: *KAF7032595.1*) derived from wheat chr-3BL. Homologous proteins satisfying query coverage > 90 % and identity > 70 % were analyzed for genetic evolution and motifs, and the results showed only crops with at least two homologous proteins (Fig. 4). The evolutionary tree was divided into three branches. The first branch contained *Triticeae dumort* (wheat, *Hordeum vulgare*, *Triticum dicoccoides* and *Aegilops tauschii*) and was divided into two subbranches (green and blue). *PmBMYD* and *RGA S-L8* were on the same (green) branch, indicating that *PmBMYD* was likely a resistance gene. Except for *CAB3489620.1*, all 41 homologous proteins from different plants had similar motif compositions and orders (Fig. 4), indicating the conservation of *PmBMYD*-encoded proteins in different plants.

## Discussion

Using the comparison of physical positions on the websites of EnsemblPlants (http://plants.ensembl.org/Triticum_aestivum/Tools/Blast) and the Triticeae Multiomics Center (https://mcgb.uestc.edu.cn/b2dsc), four QTNs in this study were found to be closely linked with previously reported genes or QTNs for PM resistance in wheat. In other words, the four QTNs are known. First, the QTN near chr02AL_AX-110,404,341 is very close to the PM resistance gene *pmHNK54* in the Henan wheat cultivar Zheng 9754. This association occurs because this marker is located between the simple sequence repeat (SSR) markers Xbarc5 and Xgwm312, which are linked to *pmHNK54* [37]. Then, the QTN near chr05AL_AX-111,769,070 is very close to the PM resistance locus *QPm.nuls-5 A*. This association occurs because this marker is located between the SSR markers Xbarc197 and Xwmc327, which are linked to *QPm.nuls-5 A* [11]. Next, the QTN near chr02BS_AX-110,111,855 is only approximately 480 kb away from the SSR marker barc55, which is linked to *pm42* [38]. Finally, the two markers chr03BL_AX-109,052,670 and chr03BL_AX-109,052,670 were not in the same LD block in the haplotype analysis of all 13 QTNs on chr-3B, although they were only 8.1 Mb apart. *Pm41* [43] and *pmHNK* [39] have been previously reported to be located on chr-3BL; *pm41* is located between the SNP markers M160 and M171, and *pmHNK* is flanked by the SSR markers Xgwm108 and Xgwm299. The genomic distance is 45 Mb between chr03BL_AX-109,052,670 and *pm41*, 8.2 Mb between chr03BL_AX-109,052,670 and *pmHNK*, 37 Mb between chr03BL_AX-111,134,486 and *pm41*, and 140 kb between chr03BL_AX-111,134,486 and *pmHNK*. This indicates that chr03BL_AX-111,134,486 should be the same locus as *pmHNK* and that chr03BL_AX-109,052,670 is a new wheat PM resistance locus on chr-3BL, which we named *Qpm-3BL*.

*Pm8* [44], *pm32* [45] and *pm39* [11] have all been reported to be located on chr-1BL. As there were no molecular markers available for anchoring *Pm8* and *pm32* on a chromosome, the two genes could not be compared with two QTNs, namely, chr01BL_AX-108,771,002 and chr01BL_AX-110,117,322. It should be noted that *pm8* and *pm32* were derived from rye and *Aegilops tauschii*, respectively, and the five resistant accessions with the elite haplotype TC were all from landraces that existed before the introduction of the two exogenous genes into common wheat; the SSR marker Xwmc719, closely linked with *pm39*, was approximately 82 Mb away from the haplotype TC. In addition, three other QTNs were found to be located on chr-1BL, namely, *QPm.osu-1B* [46] and the two linked QTNs, *QPm.vt-1BL* [47] and *QPm.vt-1B* [48], for which the closely linked SSR markers Xwmc134 and Xgwm259 were approximately 8.5 Mb and 90.0 Mb away from the haplotype TC, respectively. Therefore, the haplotype TC on chr-1BL, named *Qpm-1BL*, may be different from the above resistance genes/loci.

Although *QPm.sfr-5 A.1* [49], *QPm.ttu-5 A* [50], *Pm30* [51], *pm20* [52], *QPm.sfr-7B.1*, *QPm.sfr-7B.2* [49] and *QPm.crag-7B* [53] have been found to be located on chromosomes 5AS, 5AS, 5BS, 6BS, 7BL, 7BL and 7BL, respectively, there were no molecular markers available for anchoring them on a chromosome. Thus, these genes/loci cannot be compared with our four QTNs linked with chr05AS_AX-108,822,998, chr05BS_AX-110,494,139, chr06BS_AX-109,883,927 and chr07BL_AX-108,787,526, indicating that additional evidence is needed to determine whether the above four QTNs in this study are newly discovered QTNs.

Among the 637 accessions, 12 carried the resistance haplotype on chromosome 3BL, of which only 8 were resistant materials, namely, landraces Kumai, Baimayidan, Foshoumai, Dongmai, Jiutouniao, Wuzitoumai, Jinsita, and Bensiyuehuang. The eight materials had PM scores less than or equal to 1 in all the environments, except for Jiutouniao, which had a PM score = 2 in 2019. The other 4 susceptible accessions showed a PM score = 3 in all environments, except for Mazhamei, which had a PM score = 4 in 2017–2019. The differences in the resistance levels in the 12 accessions carrying the resistance haplotype for *Qpm-3BL* may derived from the interaction between *Qpm-3BL* and other resistance loci. We counted the distribution of 11 SNP markers (10 QTNs) that were stably detected in this study in the 12 materials. Among the 8 disease-resistant materials, 5 had 9 QTNs, and 3 had 6 QTNs. Among the 4 susceptible materials, 1 had 6 QTNs, 2 had 5 QTNs, and 1 had no resistance QTN. There were generally more resistance QTNs in the resistant materials than in the susceptible materials, which may be the reason for the difference in resistance levels among the 12 materials.

To understand the molecular mechanism of PM resistance for *Qpm-3BL*, we examined the relationship between PM resistance and gene tandem repeats. In this study, two linked genes around *Qpm-3BL*, namely, *TraesCS3B01G483600.1* and *TraesCS3B01G483700.1*, were enriched in one plant-pathogen interaction pathway, and they showed significant expression after 48 h of PM infection. More importantly, the two linked genes are homologous to *RGA S-L8*, which is related to PM resistance in barley. We compared the DNA and cDNA sequences of the two genes, and the homologies were 63.98 and 47.19 %, respectively. Interestingly, if the base T at 1528 bp (IWGSC refseqv1.0:730,284,914) of *TraesCS3B01G483600.1* was mutated to G (ending codon TAG→GAG), the cDNA sequence homology of the two genes increased to 84.25 % (> 75 %) [54]; additionally, their distance was only 22.5 kb. More interestingly, two tandem repeat genes (*TraesCS3B01G485200.1* and *TraesCS3B01G485300.1*; 23 kb) with identical sequences were found to be only 172 kb away from the *Qpm-3BL* candidate gene and identified by IWGSC RefSeqv1.0 to be NBS-LRR plant disease resistance genes. A similar association between resistance and gene tandem repeats has been reported in soybean; in other words, the copy number of genes is associated with the gene expression level [55]. Thus, PM resistance for *Qpm-3BL* may be derived from gene tandem repeats, although further evidence needs to be provided in the near future.

## Conclusions

Several substantive conclusions can be made from the results of this study, as summarized below. Among 637 core wheat accessions in Henan Province, first, 36 cultivars were found in four experimental environments to be consistently resistant to PM at the adult plant stage; in particular, 21 resistant landraces were more likely to be germplasms with previously undiscovered resistance genes, which should be fully studied and applied in wheat disease resistance breeding practice and molecular biological research. Then, eleven QTNs for PM resistance were simultaneously identified by multiple multilocus GWASs in multiple situations. Among these stable QTNs, four were previously reported, namely, *PmHNK54* [37], *QPm.nuls-5 A* [11], *Pm42* [38] and *PmHNK* [39], indicating the reliability of the results of this study. More importantly, two were newly reported in this study, namely, *Qpm-3BL* and *Qpm-1BL*. Finally, *Qpm-3BL* was validated by the different PM scores of a new KASP marker genotype in an F_2_ population. The new KASP marker is available for wheat marker-assisted breeding. Near *Qpm-3BL*, *PmBMYD* (*TraesCS3B01G483700.1*) was found by KEGG, gene expression and comparative genomic analyses to be associated with PM resistance, although its biological function and molecular mechanism need to be addressed in the future.

## Methods

### Plant materials

The genetic population in the GWAS consisted of 637 wheat core germplasms in Henan Province, including 371 ancient landraces and 266 released cultivars consisting of major large-scale popularized cultivars, fundamental parents, and current budding cultivars from the past 70 years. To validate new QTNs identified in the GWAS, we constructed an F_2_ population with 155 plants from a cross between Baimayidan (resistant) and Pumai 9 (susceptible). The abovementioned accessions and parents were provided by the Department of Molecular Breeding, Institute of Wheat, Henan Academy of Agricultural Sciences (Additional files 3 and 5).

### Disease assessment

All 637 wheat accessions were planted in the Henan Modern Agricultural Research and Development Base in Yuanyang County, Henan Province. Each accession had two rows that were one meter length, with one inch per grain. We used a combination of natural infestation and artificial inoculation in the field. In early March each year from 2017 to 2020, a strain consortium of popular physiological races provided by the Institute of Plant Protection of Henan Academy of Agricultural Sciences in Henan Province was used to inoculate all 637 wheat accessions. Inoculation, lasting for approximately 2 h each time, was performed after wheat leaf spraying and repeated after 2 days. When the susceptible control cultivar Zhoumai 19 near the heading stage was fully infected in late April or early May, the PM scores of the upper four wheat leaves for all the accessions were evaluated based on the modified five-level classification of Shi [56] (Additional file 2: Figure S6, Additional file 1: Table S4) as follows: immunity (0): the whole tiller was disease-free; high resistance (1): the flag leaf and second leaf had an incidence of less than 1 %, and the lower leaves had an incidence of less than 5 %; medium resistance (2): the flag leaf and second leaf had an incidence of 1–5 %, and 5–25 % of the lower two leaves showed severe disease; medium susceptibility (3): the flag leaf and second leaf had an incidence of 5–25 %, and the lower leaves had an incidence of 25-50 %; and high susceptibility (4): the flag leaf and second leaf had an incidence of more than 25 %, and the lower leaves had an incidence of more than 50 %.

### Genotyping and marker screening

Total genomic DNA was extracted from fresh leaves collected from plants in the field using the CTAB method [57]. Genotyping for all 637 wheat accessions with a 660 K wheat microarray was carried out by Zhongyujin Marker Biotechnology Co., Ltd., Beijing, China (www.cgmb.com.cn). To reduce errors, four screening criteria were adopted: alleles = 2, minor allele frequency (MAF) ≥ 0.01, missing ≤ 10 % and heterozygosity ≤ 10 %. After removing the markers that did not meet the abovementioned criteria, 314,548 SNP markers were available.

### Population structure and LD analysis

Based on the above 314,548 SNP markers, the population structure of all 637 wheat accessions was determined using STRUCTURE v2.3.4 software [58]. The number of subgroups (*K*) was set from 2 to 5. In a Markov chain Monte Carlo Bayesian analysis for each *K*, the length of a Markov chain consisted of 110,000 sweeps. The first 10,000 sweeps (the burn-in period) were deleted, and thereafter, the chain was used to calculate the mean log-likelihood. This process was repeated five times, and the total average log-likelihood at fixed *K* was used. The ad hoc statistic $\Delta D$, based on the rate of change in the log-likelihood of data between successive *K* values, was used to determine the suitable value of *K* [58].

Pairwise distances among all 637 accessions were calculated based on the p-distance model, and the neighbor-joining method [59] was used to construct a phylogenetic tree. PHYLIP 3.698 software (http://evolution.gs.washington.edu/phylip.html) [60] was used for the above analysis. The phylogenetic tree was drawn using FigTree v1.4.4 software (http://tree.bio.ed.ac.uk/software/figtree/). The accuracy for the phylogenetic tree was obtained from 1,000 bootstraps.

As described by Patterson et al. [61], PCA was conducted using the sample covariance matrix$$ \mathbf{X}=\mathbf{M}{\mathbf{M}}^T/\mathrm{m} $$for all 637 accessions, where *m* = 314,548, T is matrix transposition, and${\mathbf {M}}={(d_{{ij}}^{\prime })_{n \times m}}$is the normalized genotypic information matrix ($$i=1, \cdots ,637$$;$$j=1, \cdots ,m$$). $$d_{{ij}}^{{}}$$ for the *j*th SNP genotype of the *i*th accession was defined as 0 for homozygosity of the reference allele, 1 for homozygosity, and 2 for homozygosity of the nonreference allele. PCA was implemented via PLINK-v1.07 software (http://zzz.bwh.harvard.edu/plink/download.shtml#download). LD was calculated by PopLDdecay3.41 software (https://github.com/BGI-shenzhen/PopLDdecay), and the bin file was plotted and displayed by R language.

### Multilocus GWAS

A Q matrix was calculated by STRUCTURE software [62]. The kinship (K) matrix between any pair of accessions was calculated as previously described in Wang et al. [63]. All 314,548 SNPs in the 637 wheat accessions were used to conduct GWAS for APR to PM in wheat from 2017 to 2020 and determine their BLUP values using six multilocus GWAS methods, namely, mrMLM [63], FASTmrMLM [64], FASTmrEMMA [65], pLARmEB [66], pKWmEB [67] and ISIS EM-BLASSO [68], via mrMLM v4.0 software (http://cran.r-project.org/web/packages/mrMLM/index.html) [34]. All parameters in the GWAS were set at default values. The critical thresholds of significant associations for the six methods were set as logarithm of odds (LOD) = 3 (or P-value = 2 × 10^− 4^) [69].

### Haplotype analysis

The genotype file was converted to a vcf file using TASSEL v5.0 software [70], and then the target gene candidate segment was determined by vcftools v0.1.13 software [71] according to the corresponding physical position when the LD decay distance r^2^ = 0.5 and used for haplotype analysis with Haploview4.2 software [72].

### Validation of the QTN associated with the SNP chr03BL_AX-109,052,670 in F2 plants

We used 111 bp from 55 bp upstream to 55 bp downstream of the QTN (chr03BL_AX-109,052,670) on chr-3BL from Chinese Spring (IWGSC refseq v1.0) in Triticeae Multiomics Center (http://202.194.139.32/) and submitted it to LGC Technology (Shanghai) Co., Ltd. (http://sq23242284.china.herostart.com/), which designed and synthesized the KASP primers as follows:

Forward primer: F1-GAAGGTGACCAAGTTCATGCTTTGGAGGGGCTCCGACAAT.

Forward primer: F2-GAAGGTCGGAGTCAACGGATTTTGGAGGGGCTCCGACAAC.

Reverse primer: R- TCCACCACCAGTAGCTCCACACCACTCCAG.

To validate the QTN associated with the SNP chr03BL_AX-109,052,670, one KASP marker was developed and used to scan all 155 F_2_ individuals from the cross between Baimayidan (resistant) and Pumai 9 (susceptible).

### Gene tree and motif analysis

#### Gene tree

In MEGA-X software [73], ClustalW and the neighbor-joining method were used to perform protein multisequence alignment and evolutionary tree analysis, respectively. The Bootstrap method was used to test the tree, and all other parameters were default values.

#### Motif

We searched the conserved regions in the protein sequences of homologous genes with the expectation-maximization algorithm and selected eight motifs that were 8 to 50 amino acids in length. The other parameters used the default values in meme software [74].

## Supplementary Information

**Additional file 1:** **Table S1** Thirty-six wheat accessions with stable adult plant resistance (APR) to powdery mildew (PM) from 2017 to 2020, **Table S2** Pearson’s correlation coefficients of APR to PM between pairs of years, **Table S3** Genome-wide association studies (GWASs) for APR to PM in wheat based on analysis with multilocus methods, **Table S4** Grade standard of APR to PM in wheat.

**Additional file 2:** **Figure S1** Plot of *r*^2^ against distance between a pair of single-nucleotide polymorphisms (SNPs) in 637 core wheat accessions, **Figure S2** Number of quantitative trait nucleotides (QTNs) identified by multilocus GWAS approaches and their distribution on the twenty-one chromosomes, **Figure S3** Linkage disequilibrium (LD) analysis of some linked QTNs on chromosomes 1B and 3B, **Figure S4** Heat map of real and relative expression levels of candidate genes on chromosome 3B from 0 to 72 h, **Figure S5** Protein sequence alignment of the products of the *TraesCS3B01G483600.1*, *TraesCS3B01G483700.1* and *RGA S-L8* genes, **Figure S6** Reference chart for the grade standard of APR to PM in wheat.

**Additional file 3:** Population structures of 637 wheat accessions in Henan Province.

**Additional file 4:** QTNs for adult plant resistance to powdery mildew in wheat via best linear unbiased prediction (BLUP) values using multilocus GWAS.

**Additional file 5:** Genotypes of the kompetitive allele-specific PCR (KASP) marker closely linked with *Qpm-3BL* in 155 F_2_individuals.

## Data Availability

Supporting information is available from the Wiley Online Library or from the author.

## Abbreviations

PM: Powdery mildew
APR: Adult plant resistance
QTNs: Quantitative trait nucleotides
GWAS: Genome-wide association study
LD: Linkage disequilibrium
BLUP: Best linear unbiased prediction
SNP: Single-nucleotide polymorphism
KASP: Kompetitive allele-specific PCR
MAF: Minor allele frequency
LOD: Logarithm of odds
