# Supplementary material for: Identification and validation of a novel locus, Qpm-3BL, for adult plant resistance to powdery mildew in wheat using multilocus GWAS
Source: BMC Plant Biol. 2021 Jul 30;21:357. doi: 10.1186/s12870-021-03093-4 (PMC8323325; doi:10.1186/s12870-021-03093-4)
Supplement: Supplementary file 1 — Additional file 1: Table S1 Thirty-six wheat accessions with stable adult plant resistance (APR) to powdery mildew (PM) from 2017 to 2020, Table S2 Pearson’s correlation coefficients of APR to PM between pairs of years, Table S3 Genome-wide association studies (GWASs) for APR to PM in wheat based on analysis with multilocus methods, Table S4 Grade standard of APR to PM in wheat. [file 12870_2021_3093_MOESM1_ESM.doc]

**Identification and validation of a novel locus, *Qpm-3BL*, for adult plant resistance to powdery mildew in wheat using multilocus GWAS**

**Xijun Du^1, 2^, Weigang Xu^1, 2^🖂, Chaojun Peng^2^, Chunxin Li^2^, Yu Zhang^2^ and Lin Hu^2^**

1. College of Agronomy, Northwest A&F University, Yangling Shanxi, 712100, China
2. Institute of Crop Molecular Breeding/National Engineering Laboratory of Wheat/Key Laboratory of Wheat Biology and Genetic Breeding in Central Huanghuai Area/Ministry of Agriculture/Henan Key Laboratory of Wheat Germplasm Resources Innovation and Improvement, Henan Academy of Agricultural Sciences, Zhengzhou 450002, China

**Table S1 Thirty-six wheat accessions with stable adult plant resistance (APR) to powdery mildew (PM) from 2017 to 2020**

| **ID** | **Accession** | **2017** | **2018** | **2019** | **2020** |
| --- | --- | --- | --- | --- | --- |
| **HXZ27** | **Tuhulutou** | **2** | **1.5** | **0** | **2** |
| **HXZ31** | **Sanyuehuang** | **1.5** | **0.5** | **1** | **2** |
| **HXZ50** | **Liulinger** | **2** | **0.5** | **2** | **2** |
| **HXZ146** | **Baihulu** | **2** | **2** | **2** | **1** |
| **HXZ147** | **Silengmai** | **2** | **1** | **2** | **2** |
| **HXZ151** | **Duangancao** | **1** | **0.5** | **2** | **2** |
| **HXZ155** | **Liekoucao** | **1** | **0.5** | **1.5** | **1** |
| **HXZ171** | **Kumai** | **1** | **0.5** | **1** | **1** |
| **HXZ174** | **Baimayidan** | **1** | **1** | **0.5** | **1** |
| **HXZ214** | **Foshoumai** | **0.5** | **1** | **1** | **0.5** |
| **HXZ215** | **Dongmai** | **0.5** | **0.5** | **1** | **1** |
| **HXZ216** | **Jiutouniao** | **0.5** | **1** | **2** | **1** |
| **HXZ217** | **Wuzitoumai** | **0.5** | **0.5** | **0.5** | **0.5** |
| **HXZ218** | **Jinsita** | **1** | **0.5** | **1** | **1** |
| **HXZ257** | **Baigega** | **0.5** | **2** | **1** | **1** |
| **HXZ260** | **Huomai** | **1** | **1** | **2** | **2** |
| **HXZ261** | **Bensiyuehuang** | **0.5** | **1** | **0** | **1** |
| **HXZ349** | **Hongheshangtou** | **0.5** | **0.5** | **1** | **2** |
| **HXZ351** | **Hongyouzitou** | **1** | **1** | **2** | **2** |
| **HXZ352** | **Hongsuimai** | **2** | **2** | **2** | **2** |
| **HXZ353** | **Yuanzicao** | **2** | **2** | **2** | **2** |
| **HXZ437** | **Zhengtaiyu 1** | **2** | **2** | **2** | **2** |
| **HXZ465** | **Yumai 51** | **2** | **2** | **2** | **2** |
| **HXZ467** | **Yumai 54** | **1.5** | **2** | **2** | **2** |
| **HXZ482** | **Zhongyu 5** | **2** | **2** | **2** | **2** |
| **HXZ483** | **Zhongyu 6** | **2** | **2** | **2** | **2** |
| **HXZ502** | **Xinmai 13** | **2** | **2** | **2** | **1** |
| **HXZ541** | **04 zhong 36** | **1** | **1.5** | **1** | **2** |
| **HXZ542** | **Zhongyu 12** | **2** | **2** | **2** | **2** |
| **HXZ546** | **Jimai 22** | **1** | **0.5** | **2** | **1.5** |
| **HXZ557** | **Zhongluotiegan** | **1** | **2** | **1** | **2** |
| **HXZ566** | **Yulong 1325** | **2** | **2** | **2** | **2** |
| **HXZ568** | **Zhengmai 1354** | **0.5** | **0.5** | **2** | **1.5** |
| **HXZ583** | **Zhengmai 883** | **0.5** | **0.5** | **0** | **0** |
| **HXZ615** | **Guomai 0319** | **1** | **1** | **2** | **1** |
| **HXZ620** | **Zhengmai 119** | **1** | **1** | **2** | **2** |

The accessions indicated in red text are landraces, while the accessions indicated in blue text are released cultivars

**Table S2 Pearson’s correlation coefficients of APR to PM between pairs of years**

|  | **2017** | **2018** | **2019** | **2020** | **BLUP ^a^** |
| --- | --- | --- | --- | --- | --- |
| **2017** | **1** | **0.9044**** | **0.8472**** | **0.6387**** | **0.9433**** |
| **2018** | **0.9044**** | **1** | **0.8915**** | **0.6489**** | **0.9569**** |
| **2019** | **0.8472**** | **0.8915**** | **1** | **0.6258**** | **0.9300**** |
| **2020** | **0.6387**** | **0.6489**** | **0.6258**** | **1** | **0.7896**** |
| **BLUP** | **0.9433**** | **0.9569**** | **0.9300**** | **0.7896**** | **1** |

^a^ BLUP: best linear unbiased prediction; **: significance at the 0.01 probability level

**Table S3** Genome-wide association studies (GWASs) for APR to PM in wheat based on analysis with multilocus methods

| **Associated SNP** | **Chr** | **Position (bp)** | **LOD score ^a^** | **r^2^ (%)** | **Repeatability ^b^**  **(times)** | **Year** | **No. of methods** | **R/S allele** |
| --- | --- | --- | --- | --- | --- | --- | --- | --- |
| **AX-110990914** | **1A** | **513878783** | **3.40-5.62** | **1.02-1.39** | **2** | **2019** | **2** | **A/C** |
| **AX-109297204** | **1A** | **579020464** | **3.07-9.67** | **0.85-7.11** | **5** | **2020** | **5** | **C/T** |
| **AX-9516160** | **1A** | **589446126** | **3.14-3.43** | **2.14-2.58** | **2** | **2017, 2019** | **1** | **T/C** |
| **AX-108767843** | **2A** | **677522478** | **4.60** | **0** | **1** | **2019** | **1** | **C/T** |
| **AX-110404341** | **2A** | **694749410** | **3.54-8.02** | **2.25-4.66** | **6** | **2017, 2019** | **3** | **T/G** |
| **AX-110940978** | **2A** | **694842599** | **4.08** | **2.91** | **1** | **2019** | **1** | **C/A** |
| **AX-108963034** | **2A** | **762503674** | **3.39** | **1.96** | **1** | **2017** | **1** | **C/T** |
| **AX-110914420** | **3A** | **15348646** | **3.03** | **0.13** | **1** | **2017** | **1** | **G/C** |
| **AX-110495706** | **3A** | **683519275** | **5.91** | **1.11** | **1** | **2020** | **1** | **G/A** |
| **AX-109293922** | **4A** | **625246774** | **3.63** | **2.28** | **1** | **2017** | **1** | **T/G** |
| **AX-110958418** | **4A** | **665504629** | **3.03-4.81** | **0.42-2.39** | **2** | **2017** | **2** | **T/C** |
| **AX-111566246** | **4A** | **708587290** | **3.41-3.96** | **1.70-2.07** | **2** | **2019** | **2** | **T/C** |
| **AX-110945970** | **5A** | **3359216** | **6.02** | **0.37** | **1** | **2020** | **1** | **T/C** |
| **AX-111623495** | **5A** | **5681704** | **3.03** | **1.02** | **1** | **2017** | **1** | **A/G** |
| **AX-111679487** | **5A** | **480787120** | **4.07** | **0.28** | **1** | **2020** | **1** | **C/T** |
| **AX-108822998** | **5A** | **5704663** | **3.21-6.43** | **1.13-3.68** | **5** | **2018-2020** | **3** | **T/C** |
| **AX-111769070** | **5A** | **502896109** | **3.11-4.40** | **1.58-2.19** | **3** | **2017-2018** | **3** | **C/G** |
| **AX-110000215** | **5A** | **503787441** | **3.56** | **0.19** | **1** | **2018** | **1** | **G/A** |
| **AX-109308419** | **5A** | **503799476** | **3.30** | **3.08** | **1** | **2018** | **1** | **C/T** |
| **AX-109934491** | **5A** | **618845609** | **3.59** | **1.71** | **1** | **2019** | **1** | **C/T** |
| **AX-109379710** | **5A** | **618871152** | **8.36** | **3.22** | **1** | **2018** | **1** | **C/T** |
| **AX-109324435** | **5A** | **619484040** | **4.18** | **0.49** | **1** | **2017** | **1** | **G/A** |
| **AX-110623345** | **6A** | **38462136** | **5.15** | **1.49** | **1** | **2018** | **1** | **A/G** |
| **AX-108798074** | **6A** | **1897659** | **4.43** | **0.30** | **1** | **2020** | **1** | **G/A** |
| **AX-110523644** | **7A** | **26936652** | **4.28-4.42** | **1.43-1.63** | **2** | **2020** | **2** | **C/G** |
| **AX-111021260** | **7A** | **41817054** | **3.76** | **2.78** | **1** | **2019** | **1** | **A/G** |
| **AX-111096680** | **7A** | **276183551** | **4.59-6.03** | **1.21-2.46** | **3** | **2017-2018** | **3** | **A/G** |
| **AX-111291315** | **7A** | **611241100** | **3.55** | **1.33** | **1** | **2018** | **1** | **G/A** |
| **AX-110088924** | **7A** | **688686360** | **4.15** | **0.31** | **1** | **2019** | **1** | **A/G** |
| **AX-108771002** | **1B** | **582441405** | **3.73-3.90** | **0-2.13** | **2** | **2018-2019** | **2** | **C/T** |
| **AX-110117322** | **1B** | **582542111** | **3.52-8.37** | **1.27-3.35** | **6** | **2017-2019** | **4** | **C/A** |
| **AX-111575864** | **1B** | **655829591** | **4.51** | **2.64** | **1** | **2019** | **1** | **G/A** |
| **AX-111613047** | **1B** | **655829899** | **3.55-6.11** | **2.24-2.53** | **2** | **2018** | **2** | **A/G** |
| **AX-108771080** | **2B** | **25602184** | **3.72** | **2.52** | **1** | **2018** | **1** | **G/A** |
| **AX-110111855** | **2B** | **26097938** | **3.37-5.82** | **1.07-3.04** | **6** | **2017-2018** | **4** | **G/A** |
| **AX-111681029** | **2B** | **534862167** | **3.14** | **2.02** | **1** | **2020** | **1** | **C/T** |
| **AX-112286960** | **2B** | **655007845** | **3.55** | **0.13** | **1** | **2020** | **1** | **T/C** |
| **AX-108784950** | **2B** | **690667026** | **3.78-3.95** | **3.69-3.84** | **2** | **2017-2018** | **2** | **C/G** |
| **AX-110609772** | **2B** | **707657133** | **4.91** | **2.19** | **1** | **2018** | **1** | **C/T** |
| **AX-108832292** | **2B** | **710054149** | **5.38** | **0** | **1** | **2019** | **1** | **T/C** |
| **AX-110403216** | **2B** | **731475473** | **5.91** | **1.06** | **1** | **2017** | **1** | **A/G** |
| **AX-110471573** | **2B** | **753837087** | **4.66-5.40** | **1.42-1.71** | **2** | **2017-2018** | **2** | **C/A** |
| **AX-111728382** | **2B** | **793799190** | **4.86-6.28** | **0.27-1.70** | **2** | **2020** | **2** | **G/C** |
| **AX-110087544** | **3B** | **22773066** | **3.20-5.69** | **1.25-2.19** | **3** | **2017-2018** | **2** | **C/A** |
| **AX-109914478** | **3B** | **23723086** | **3.65** | **1.36** | **1** | **2018** | **1** | **G/C** |
| **AX-108740220** | **3B** | **52731608** | **4.47** | **0.64** | **1** | **2017** | **1** | **G/A** |
| **AX-110669258** | **3B** | **360892138** | **3.15** | **2.45** | **1** | **2020** | **1** | **T/C** |
| **AX-110509313** | **3B** | **436568524** | **5.02** | **0** | **1** | **2018** | **1** | **T/C** |
| **AX-109052670** | **3B** | **730604593** | **3.22-20.18** | **0-12.98** | **12** | **2017-2020** | **5** | **T/C** |
| **AX-108964753** | **3B** | **738503284** | **5.17** | **5.84** | **1** | **2017** | **1** | **T/C** |
| **AX-111134486** | **3B** | **738687251** | **4.12-4.70** | **1.64-1.99** | **2** | **2017-2018** | **2** | **C/T** |
| **AX-109500886** | **3B** | **739032293** | **5.75** | **2.18** | **1** | **2019** | **1** | **A/G** |
| **AX-110909724** | **3B** | **739164356** | **4.28** | **1.71** | **1** | **2019** | **1** | **T/C** |
| **AX-109382105** | **3B** | **773018315** | **3.05-3.50** | **1.40-4.50** | **2** | **2017-2018** | **2** | **T/G** |
| **AX-108796244** | **3B** | **788770310** | **3.43-3.48** | **1.76-1.84** | **2** | **2019** | **2** | **A/G** |
| **AX-111238474** | **3B** | **813114323** | **3.81-5.12** | **3.04-3.22** | **2** | **2019** | **2** | **T/A** |
| **AX-110694983** | **4B** | **171316602** | **4.97-5.74** | **1.46-3.87** | **2** | **2018** | **2** | **T/C** |
| **AX-111489069** | **4B** | **568448485** | **6.39** | **1.00** | **1** | **2017** | **1** | **A/T** |
| **AX-110956447** | **4B** | **640644066** | **3.72** | **1.40** | **1** | **2018** | **1** | **T/C** |
| **AX-108990595** | **5B** | **27300179** | **3.55** | **1.65** | **1** | **2017** | **1** | **C/A** |
| **AX-110494139** | **5B** | **27356806** | **3.28-3.84** | **0.39-2.00** | **3** | **2017, 2019** | **2** | **G/T** |
| **AX-111086850** | **5B** | **64740489** | **4.67** | **0.61** | **1** | **2017** | **1** | **G/C** |
| **AX-109386068** | **5B** | **191674162** | **3.01** | **0.52** | **1** | **2017** | **1** | **A/G** |
| **AX-95166572** | **5B** | **268502541** | **4.17** | **3.90** | **1** | **2018** | **1** | **T/G** |
| **AX-111049524** | **5B** | **661309235** | **3.87** | **5.61** | **1** | **2019** | **1** | **T/C** |
| **AX-111583119** | **5B** | **665570739** | **3.63** | **0.54** | **1** | **2017** | **1** | **T/C** |
| **AX-109470391** | **5B** | **681189731** | **3.90** | **1.72** | **1** | **2019** | **1** | **A/G** |
| **AX-109883927** | **6B** | **159311957** | **4.04-5.99** | **1.42-2.81** | **3** | **2017-2019** | **2** | **C/A** |
| **AX-111462467** | **6B** | **259305876** | **3.72** | **4.25** | **1** | **2019** | **1** | **A/G** |
| **AX-111676103** | **6B** | **277631904** | **3.06-3.79** | **1.07-1.41** | **2** | **2017** | **2** | **T/C** |
| **AX-109469971** | **6B** | **368197590** | **4.07** | **3.08** | **1** | **2020** | **1** | **C/T** |
| **AX-109825912** | **6B** | **714086991** | **3.18** | **0.45** | **1** | **2017** | **1** | **G/C** |
| **AX-110955530** | **7B** | **1208389** | **4.46** | **1.27** | **1** | **2019** | **1** | **C/T** |
| **AX-110027919** | **7B** | **47342576** | **3.44** | **0** | **1** | **2018** | **1** | **G/A** |
| **AX-94710185** | **7B** | **251136521** | **5.29** | **1.05** | **1** | **2017** | **1** | **T/C** |
| **AX-108787526** | **7B** | **405972463** | **3.24-9.91** | **1.76-4.46** | **6** | **2018-2020** | **4** | **G/A** |
| **AX-108768717** | **7B** | **566473938** | **3.38** | **0.69** | **1** | **2017** | **1** | **T/C** |
| **AX-110963653** | **7B** | **586966363** | **3.01** | **1.33** | **1** | **2019** | **1** | **A/G** |
| **AX-111587257** | **7B** | **654863276** | **3.20** | **0.80** | **1** | **2018** | **1** | **G/C** |
| **AX-109041223** | **7B** | **656524287** | **4.42** | **1.37** | **1** | **2017** | **1** | **A/G** |
| **AX-89752531** | **7B** | **708445015** | **4.00** | **0** | **1** | **2020** | **1** | **A/G** |
| **AX-111895014** | **1D** | **92736364** | **3.46** | **0.74** | **1** | **2017** | **1** | **A/G** |
| **AX-109270221** | **1D** | **475654058** | **3.48** | **0.83** | **1** | **2018** | **1** | **A/T** |
| **AX-112286211** | **1D** | **423606495** | **3.06** | **1.41** | **1** | **2019** | **1** | **T/G** |
| **AX-111629055** | **1D** | **18097556** | **4.58** | **0.31** | **1** | **2020** | **1** | **A/C** |
| **AX-109451047** | **2D** | **66856217** | **6.21** | **2.74** | **1** | **2017** | **1** | **C/G** |
| **AX-109825170** | **2D** | **639666367** | **6.07** | **2.69** | **1** | **2017** | **1** | **T/C** |
| **AX-110483370** | **2D** | **607520008** | **3.54** | **0** | **1** | **2018** | **1** | **C/G** |
| **AX-110417313** | **2D** | **640894029** | **3.13** | **1.98** | **1** | **2018** | **1** | **T/C** |
| **AX-111988009** | **2D** | **69269361** | **3.88-5.87** | **0.18-1.89** | **3** | **2020** | **3** | **T/C** |
| **AX-109999625** | **3D** | **464166622** | **3.44-4.21** | **1.39-3.69** | **2** | **2020** | **2** | **G/A** |
| **AX-110599821** | **3D** | **472449022** | **3.73-4.01** | **0.27-2.34** | **2** | **2020** | **2** | **A/G** |
| **AX-109852738** | **3D** | **550193002** | **5.13-9.16** | **0.52-4.89** | **4** | **2020** | **4** | **C/T** |
| **AX-94896513** | **5D** | **550441953** | **4.93** | **2.34** | **1** | **2017** | **1** | **C/T** |
| **AX-111702691** | **5D** | **370471528** | **7.23** | **2.68** | **1** | **2018** | **1** | **C/T** |
| **AX-109954383** | **5D** | **471830841** | **6.05** | **0.85** | **1** | **2018** | **1** | **G/A** |
| **AX-111559585** | **5D** | **29352040** | **3.49-7.68** | **0.34-4.39** | **6** | **2020** | **6** | **G/T** |
| **AX-108910713** | **5D** | **486935936** | **4.33-11.08** | **0.84-7.05** | **5** | **2020** | **5** | **T/C** |
| **AX-110527938** | **7D** | **2608577** | **3.05-5.30** | **1.29-3.75** | **3** | **2018** | **3** | **C/T** |
| **AX-108817689** | **7D** | **111750009** | **4.14** | **1.95** | **1** | **2020** | **1** | **C/T** |

^a^ LOD: logarithm of odds; ^b^ Repeatability: the number of times in which the QTN was identified by various multilocus genome-wide association study approaches across various years, where these approaches were mrMLM, FASTmrMLM, FASTmrEMMA, ISIS EM-BLASSO, pLARmEB and pKWmEB.

**Table S4** Grade standard of APR to PM in wheat

| **Score** | **Leaves 1-2 (%)** | **Leaves 3-4 (%)** |
| --- | --- | --- |
| 0 | 0 | 0 |
| 1 | < 1 | < 5 |
| 2 | 1-5 | 5-25 |
| 3 | 5-25 | 25-50 |
| 4 | > 25 | > 50 |
