# Supplementary material for: Identification and validation of a novel locus, Qpm-3BL, for adult plant resistance to powdery mildew in wheat using multilocus GWAS
Source: BMC Plant Biol. 2021 Jul 30;21:357. doi: 10.1186/s12870-021-03093-4 (PMC8323325; doi:10.1186/s12870-021-03093-4)
Supplement: Supplementary file 2 — Additional file 2: Figure S1 Plot of r2 against distance between a pair of single-nucleotide polymorphisms (SNPs) in 637 core wheat accessions, Figure S2 Number of quantitative trait nucleotides (QTNs) identified by multilocus GWAS approaches and their distribution on the twenty-one chromosomes, Figure S3 Linkage disequilibrium (LD) analysis of some linked QTNs on chromosomes 1B and 3B, Figure S4 Heat map of real and relative expression levels of candidate genes on chromosome 3B from 0 to 72 h, Figure S5 Protein sequence alignment of the products of the TraesCS3B01G483600.1, TraesCS3B01G483700.1 and RGA S-L8 genes, Figure S6 Reference chart for the grade standard of APR to PM in wheat. [file 12870_2021_3093_MOESM2_ESM.doc]

**Identification and validation of a novel locus, *Qpm-3BL*, for adult plant resistance to powdery mildew in wheat using multilocus GWAS**

**Xijun Du^1, 2^, Weigang Xu^1, 2^🖂, Chaojun Peng^2^, Chunxin Li^2^, Yu Zhang^2^ and Lin Hu^2^**

1. College of Agronomy, Northwest A&F University, Yangling Shanxi, 712100, China
2. Institute of Crop Molecular Breeding/National Engineering Laboratory of Wheat/Key Laboratory of Wheat Biology and Genetic Breeding in Central Huanghuai Area/Ministry of Agriculture/Henan Key Laboratory of Wheat Germplasm Resources Innovation and Improvement, Henan Academy of Agricultural Sciences, Zhengzhou 450002, China


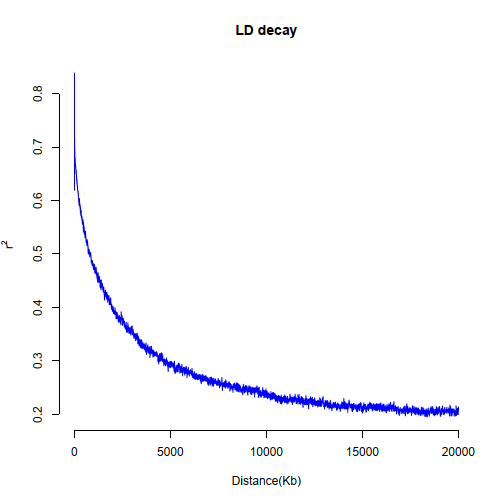


**Figure S1** Plot of *r*^2^ against distance between a pair of single-nucleotide polymorphisms (SNPs) in 637 core wheat accessions


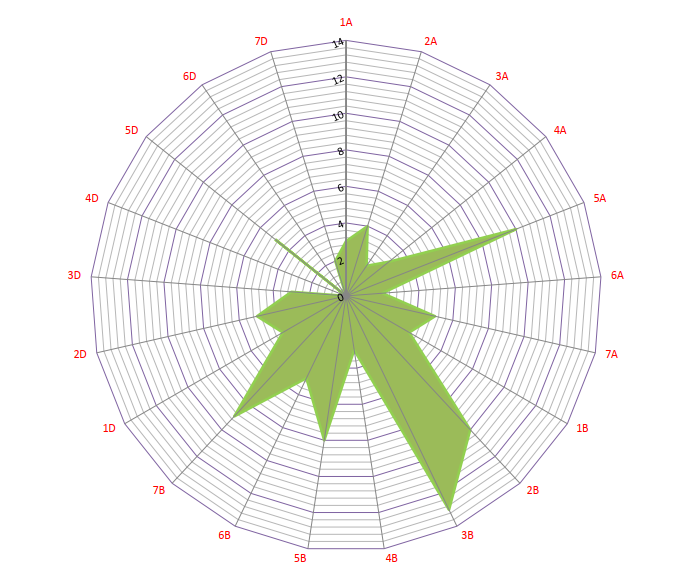


**Figure S2** Number of quantitative trait nucleotides (QTNs) identified by multilocus GWAS approaches and their distribution on the twenty-one chromosomes

**a**  **b**

**
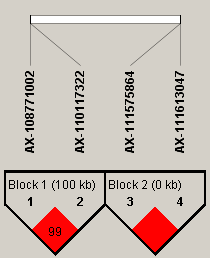
** **
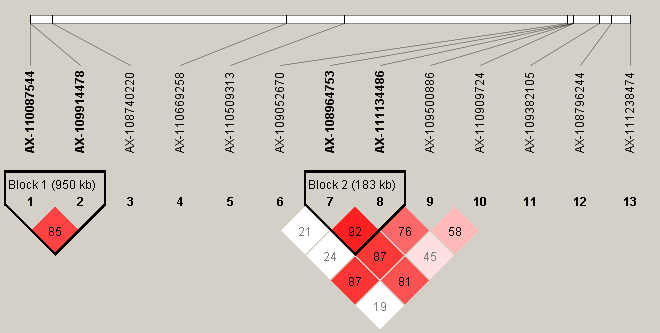
**

**Figure S3** Linkage disequilibrium (LD) analysis of some linked QTNs on chromosomes 1B and 3B


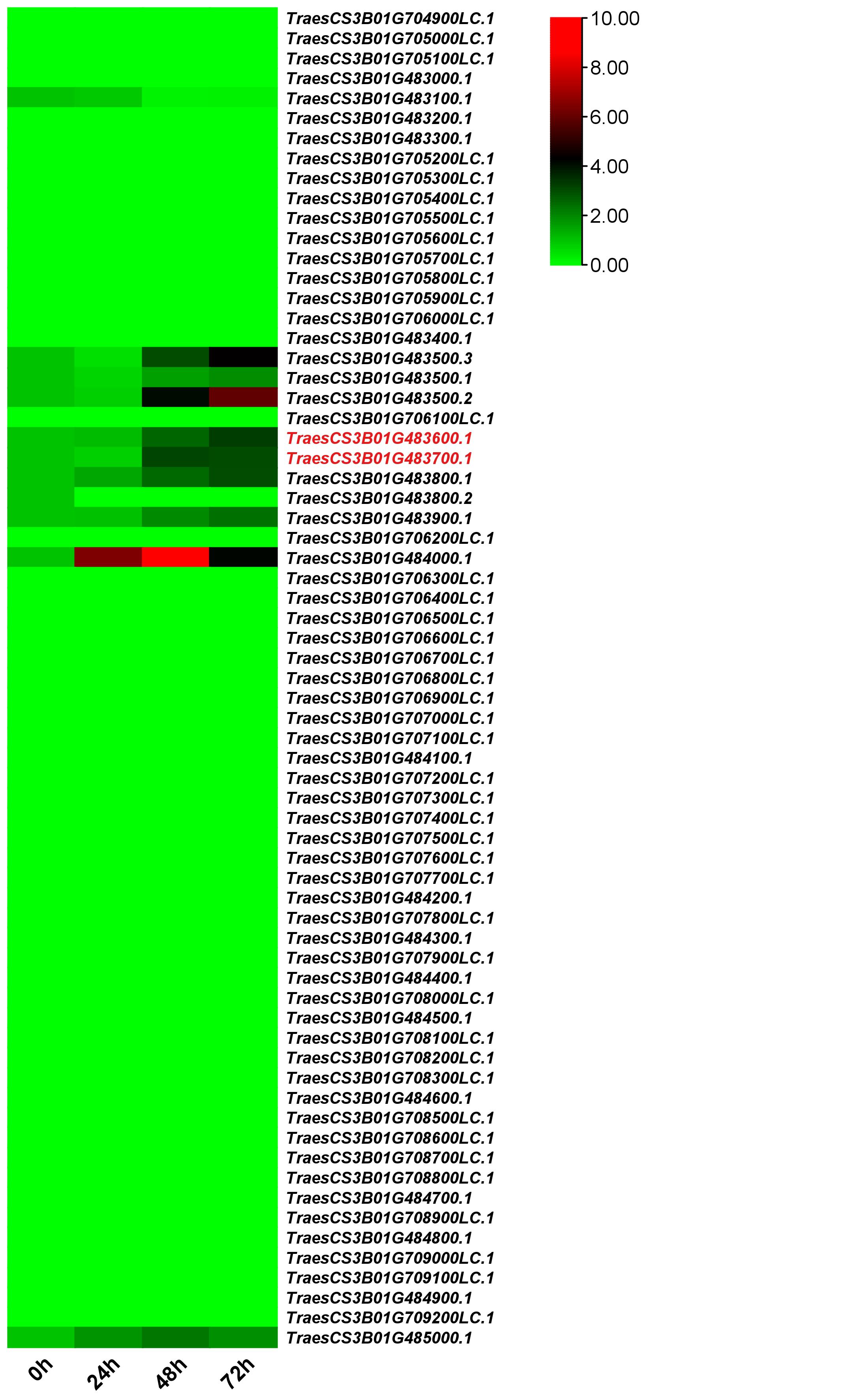


**Figure S4** Heat map of real and relative expression levels of candidate genes on chromosome 3B from 0 to 72 h. No expression at 0, 24, 48 and 72 h is indicated by zero, while relative expression levels at 24, 48 and 72 h for the other genes were calculated from their corresponding real expression levels at 0 h.

**Figure S5** Protein sequence alignment of the products of the *TraesCS3B01G483600.1*, *TraesCS3B01G483700.1* and *RGA S-L8* genes


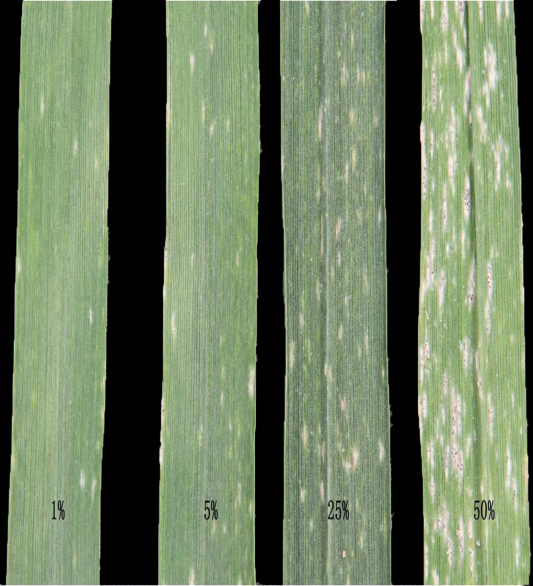


**Figure S6** Reference chart for the grade standard of APR to PM in wheat
